# To play or not to play, that is the question: an interview study with amateur football coaches on perceptions of pain during sports participation

**DOI:** 10.1136/bmjsem-2024-001941

**Published:** 2024-07-09

**Authors:** Sofi Sonesson, Hanna Lindblom, Martin Hägglund

**Affiliations:** 1 Department of Health, Medicine and Caring Sciences, Unit of Physiotherapy, Linköping University, Linköping, Sweden

**Keywords:** Football, Qualitative Research, Prevention, Sports & exercise medicine, Sports medicine

## Abstract

Amateur football coaches play a key role in preventing, assessing and treating pain among their players, as they are often the first point of contact and may be the main source of advice and guidance. The objective of this study was to explore amateur football coaches’ perceptions of pain during sports participation and their approach to pain management. We conducted a qualitative interview study with 20 amateur football coaches from a strategically selected sample of male and female, and junior and senior teams. A semistructured interview guide and conventional qualitative content analysis were used. One overall theme emerged: *To play or not to play—coaches navigating difficult terrain with limited resources*. The theme included four main categories: *How can pain be understood?; Can pain be avoided?; How to manage players with pain?; What resources do we need?* Different ways of understanding pain emerged, and coaches described that players have different pain thresholds. Pain was seen as a part of the game that cannot be completely avoided. In general, there was a restrictive attitude regarding pain medication, though actual consumption was not known. Coaches emphasised shared responsibility with players to achieve adequate training loads, a positive communication climate surrounding pain, and a need for education and competence. In conclusion, coaches expressed various interpretations of pain during sports participation and pain management, where they need to take on great responsibility despite limited medical competence. Coaches believed that adequate pain management is important, and their keys to reducing the risk of pain included structured and customised training, a well-balanced training load and recovery, and a positive communication climate in the team. Coaches often decide whether players experiencing pain can participate in team training and match play, emphasising the need for education support and access to medical competence.

**WHAT IS ALREADY KNOWN ON THIS TOPIC**

- Pain during sports participation is common in all levels and sports disciplines.
- Football involves a high risk of injury and pain, and players often continue to play despite the pain, which could potentially increase the risk of a time-loss injury.
- There is a growing body of research on pain management in professional sports, while there is a lack of studies on how amateur coaches perceive and manage pain.

**WHAT THIS STUDY ADDS**

- Amateur football coaches must assume great responsibility in pain management with their players despite limited medical competence.
- Coaches highlighted a need for education and support for coaches, and access to proper medical competence for their players.
- Coaches’ keys to reducing the risk of pain included structured and customised training, a well-balanced training load and recovery and a positive communication climate in the team.
- Coaches pointed out important responsibilities of their players, such as participation in team training to achieve sufficient training loads and fitness; self-care, including physical training and rehabilitation; sleep; nutrition and recovery; and communication with their coaches.

**HOW THIS STUDY MIGHT AFFECT RESEARCH, PRACTICE OR POLICY**

- Amateur football coaches need education and support to work strategically to prevent pain among players and to be able to manage players with pain. Educational efforts and guidelines for pain management should be provided in all clubs. Access to medical expertise is crucial for effectively managing pain during sports participation.
- Emphasising a positive communication climate between coaches and players is crucial to encouraging players to report pain confidently.
- Player education could include interventions to promote self-care (eg, taking responsibility for being fit to play by performing strength and conditioning and adhering to rehabilitation protocols) and wellness factors (eg, sleep, nutrition and recovery).

## Introduction

 Pain during sports participation is common in all levels and disciplines.^1^,^3^ Football is one of the most widely played sports and involves a high risk of injury and pain.^4^,^7^ Pain during sports participation may have multidimensional causes, affecting the athlete physically, psychologically and socially.^8 9^ Social structures and cultural norms within sports may imply that pain is a natural part of an athlete’s identity,^10^ and athletes may continue to play despite pain.^11^ However, playing football with a physical complaint can increase the risk of a time-loss injury.^12^ Pain management is crucial for athletes’ performance, recovery and well-being,^13^ and encompasses knowledge, attitudes and practices.^9^

While there is a growing body of research on pain management in professional sports,^13^,^16^ there is a lack of studies in amateur sports. In professional sports, sports medicine clinicians have a key role in pain management,^13^ while in amateur-level sports, access to medical support is limited. Amateur coaches often are unpaid volunteers with varying levels of education and experience. Motivations, goals and challenges may differ between professional and amateur levels. Therefore, it is important to understand perceptions of pain and pain management in amateur sports. Amateur football coaches play a key role in preventing, assessing and treating pain among their players, as they are often the first point of contact and may be the main source of advice and guidance. However, little is known about how these coaches perceive and manage pain and what factors affect their pain management practices. To address this gap, this paper explored amateur football coaches’ perceptions of pain during sports participation and their approach to pain management. The concept of pain during sports participation embraces all types of pain that may be present during football play, regardless of the presence or absence of injury.

## Methods

### Study design

This qualitative study involved individual interviews with football coaches within one regional Swedish football district (Östergötland). We used a semistructured interview guide (online supplemental appendix 1), including two topics: (1) perceptions of pain during football and (2) experiences of and support for injury prevention training. The present paper focuses on pain during football. The manuscript has been checked against the Consolidated criteria for reporting qualitative research checklist.^17^

### Participants and inclusion

Football coaches for players ≥14 years within one football district (containing approximately 100 clubs and 900 coaches for the target group) were eligible. Another inclusion criterion was the experience of the injury prevention exercise programmes *Knee Control*/*Knee Control+* to be able to respond to the questions relating to this topic (analysed separately and presented elsewhere). Participating coaches were strategically selected, aiming for maximum variation regarding player age (junior/senior), sex, playing level and geographical area (urban/rural). Based on our previous experience and the literature,^18^ we estimated that 20 interviews with coaches representing different perspectives would be sufficient to respond to the study’s aim. First, we sent an email with study information to strategically selected coaches (n=50), followed by telephone contact until 20 agreed to participate. In total, we contacted 30 coaches, 10 of whom declined to participate due to lack of interest, no longer coaching or having no experience with the *Knee Control* programmes. After completing 20 interviews, rich and comprehensive data were captured, and no new information was forthcoming in the last few interviews. Therefore, we did not proceed with further recruitment. Regarding the participants' knowledge of the interviewer, most respondents had no contact with the researchers before the interviews. Still, three coaches had taken part in previous studies by the research group.

### Data collection

Each interview was conducted by telephone by one of two female sports physiotherapists (HL and SS), both PhDs, with previous experience conducting qualitative studies. Interviews took place from January to March 2023 and were recorded using a Dictaphone. Before the interview, the interviewer presented herself by name, introduced herself as a researcher and explained the aim of the study. Oral or written informed consent was collected from all participants before the interview.

The interview guide included questions about experiences of pain during football, pain management strategies, thoughts about prevention of pain and attitudes regarding pain medication. This study explores amateur football coaches’ perceptions of pain during sports participation, embracing all types of pain that may be present during football play, including pain related to acute and gradual-onset injury and overload, as well as other types of pain, such as menstrual pain and headaches (online supplemental appendix 1). The interview guide was constructed by the researchers (SS, HL, MH) and was tested in two pilot interviews. After the pilot interviews, we concluded that the interview guide was sufficient for the study aim, no changes were made and both pilot interviews were included in the analysis. After completing 20 interviews, the recorded material was transcribed verbatim by a licensed transcription service and then analysed. Transcripts or interpretations were not returned to the coaches for comments.

### Analysis

Inductive qualitative content analysis of the transcribed interviews was employed, according to Hsieh and Shannon.^19^ The interview transcripts were read and reread repeatedly to obtain an overall picture. Both SS and HL read the transcripts thoroughly. Analyses were performed using NVivo (R V.14.23.2). Each interview transcript was imported into the NVivo software. The coding process started by marking meaning units that addressed the study aim and the meaning units were given codes that illustrated their content. Codes were sorted into preliminary subcategories and main categories that were internally homogeneous and externally heterogeneous. SS analysed the first interview, which was discussed with HL and MH. After that, SS analysed 10 interviews and made a preliminary categorisation, which was discussed with HL and MH before the last 10 interviews were analysed. New information was categorised into existing categories, when appropriate, or categorised into new categories. Main categories and subcategories were continuously refined and reorganised to represent the data. After analysing and categorising all data, all authors discussed the analysis and categorisation, and minor changes were made to reach a consensus. During the analysis process, the authors checked the transcripts to ascertain that the analysis and interpretations were grounded in the data obtained from the interviews. One author (MH) was not directly involved in the data collection process but had extensive experience in football research and coaching, thus providing an external perspective during the analysis. All authors agreed on the final main categories and subcategories. Finally, a negative case analysis was performed, where information within each category was critically revised to ensure all codes were correctly categorised.

### Patient and public involvement

This research was conducted without patient or public involvement. The research question and the interview guide were inspired by dialogue with coaches participating in workshops and by player responses to surveys about football-related pain in our previous studies.^7^

## Results

The 20 interviews lasted between 27 and 60 min (mean 42 min, SD 10) and included 18 male and 2 female coaches (mean age 48.4 years, SD 5.9). Participants coached adolescent or adult male players in the 3rd–9th league (out of 9 leagues) or female players in the 4th–6th league (out of 6 leagues), 9 coached male teams, 11 coached female teams, 10 were junior and 10 were senior teams. Nine of the coaches had experience coaching both male and female teams. All had (at least) basic coach education and a mean of 13 years of coach experience (range 2–45 years).

**Figure 1:**
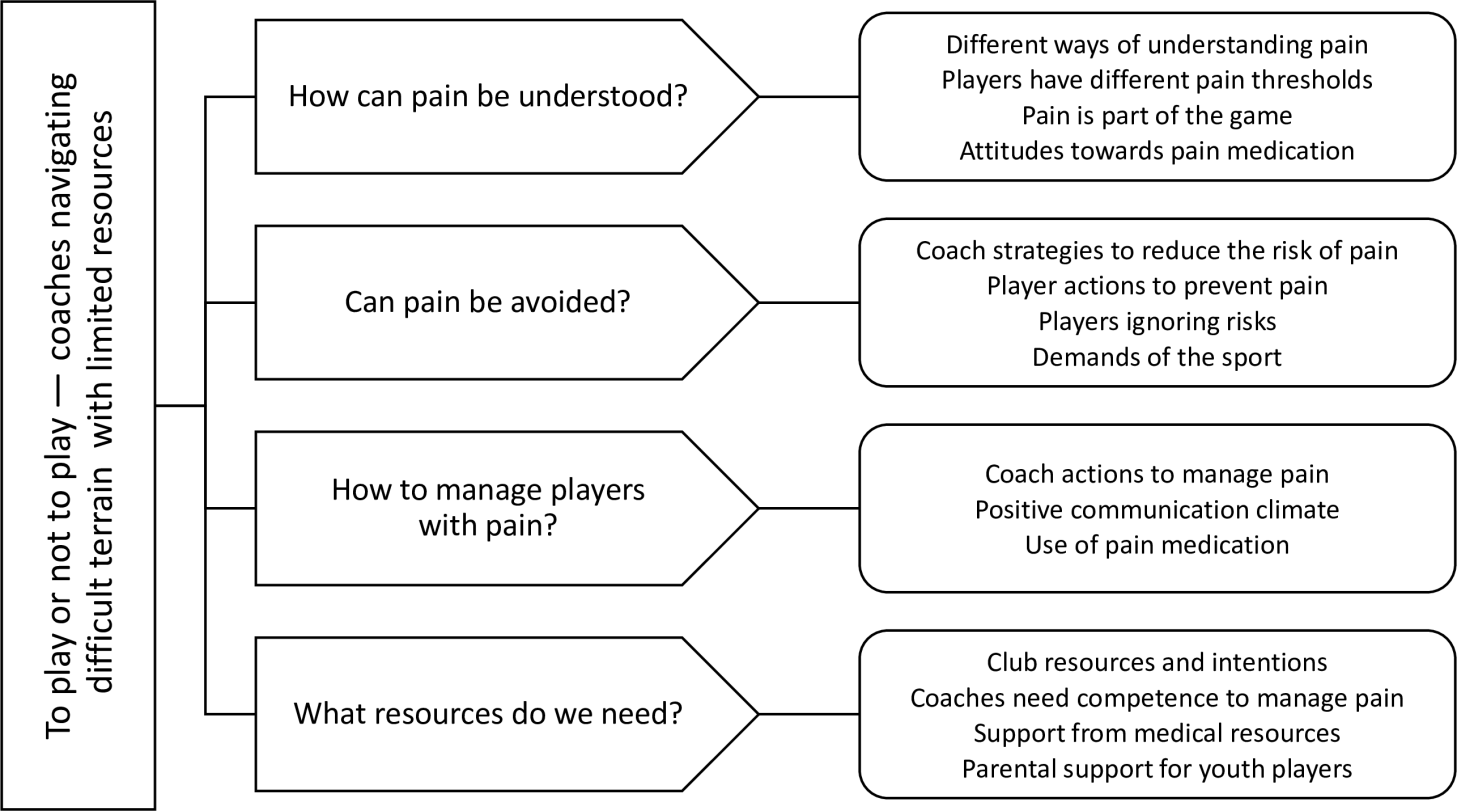
The analysis of coaches’ perceptions of pain during sports participation and approach to pain management is displayed as 1 overall theme, 4 main categories and 15 subcategories.

The results are displayed as 1 overall theme, 4 main categories and 15 subcategories. The theme described *To play or not to play—coaches navigating difficult terrain with limited resources*, which involves various interpretations of pain during sports participation and pain management, where coaches must assume great responsibility despite limited medical competence. The main categories were: (1) *How can pain be understood?;* (2) *Can pain be avoided?;* (3) *How to manage players with pain?* and (4) *What resources do we need?* (figure 1).

### How can pain be understood?

Coaches described various perceptions of pain in four subcategories: *different ways of understanding pain, players have different pain thresholds, pain is part of the game* and *attitudes towards pain medication* (table 1). The coaches described pain as a complex, common phenomenon with multifactorial causes and that pain should be interpreted as a warning sign from the body. Mainly, they related pain to a sudden or gradual onset of football-related injury. However, non-sport-related pain, such as menstrual pain and headaches related to stress in school, was also mentioned. There were views that pain can be prevented and that the absence of pain and injury is associated with better sports performance. Coaches observed pain-related anxiety and different motivations for sports in players, which influenced the players’ training and match availability and performance. They perceived that players have various pain tolerance and may tend to either hide or show pain. Coaches described that players sometimes continue to play even while experiencing pain because they do not want to appear weak or due to loyalty toward their team. Coaches reported that some players who frequently complain about pain may have alternative motives, such as attention-seeking or avoiding training. Football was perceived as a tough game where pain cannot be avoided. Hence, players need to tolerate some pain. Coaches discussed the trade-off between playing through pain, which could be beneficial in the short-term and long-term negative consequences, as well as the importance of life beyond football and not risking long-term health. Pain medication was generally discouraged, advocating proper injury treatment over masking pain. Exceptions were short-term use for sports-related pain or other conditions such as headache or menstrual pain.

**Table 1:** Examples of quotes for the four subcategories in the main category: *How can pain be understood?*

ID	Meaning unit	Subcategory
9	Some may feel pain when challenged beyond their current ability level. Some players feel energised when they are pushed to their limits, and then they perform at their best. Others may back off a bit when they feel they are not ready to take on the challenge and then psychological explanatory variables might pop up. It can be hard to say no, I don’t feel ready to play this match. It may be easier to blame knee pain.	Different ways of understanding pain
2	When it hurts, it’s a sign, a warning sign that something is not right. We need to listen to that.	
11	It varies, […] a player’s pain and ways to endure pain. If you think about exercise, for example, whether you exercise with pain or not. I have experienced players who stay home for a week when they cough once. Other players play an entire season while in pain. It’s very individual.	Players have different pain thresholds
12	They [players] are different. And if you get to know them, you'll notice what it’s like. If there is someone who never has pain who says that it was this or that, then I think you listen more carefully. Then there are those who are always in constant pain. If you cry wolf too many times, you may not really listen when it’s serious.	
17	It’s a sport we're doing. There’s no getting away from the fact that there will be tackles. […] Injuries are unfortunately a part of sports.	Pain is part of the game
15	You don't want to put your friends in trouble. […] Lower down in the divisions, it can be quite challenging to get enough players in a team together for matches.	
18	Some want to play so badly; they feel like they want to be loyal to the team and then they play even though they have pain. But our role is to sort of put the brakes on it.	Attitudes towards pain medication
11	Of course, you shouldn't play with painkillers. But if it does not do any harm and the doctor has okayed it, then I think that on rare occasions it is okay to play with painkillers. But I know too little about it.	

ID refers to the coach ID within the study. Some quotations were shortened, which is indicated by […].

### Can pain be avoided?

Coaches described factors affecting the risk of pain and strategies to prevent pain in four subcategories: *coach strategies to reduce the risk of pain, player actions to prevent pain, players ignoring risks* and *demands of the sport* (table 2). Coaches structured and adapted the training to their group of players, that is, based on players’ sex, age/maturity, level of play, and the status and specific needs of individual players. They discussed the optimal balance between pushing their players to participate in team training and overloading them. Suggested player responsibilities included team and individual training to improve fitness and muscle strength and adhere to warm-ups, injury prevention and rehabilitation exercises. Coaches emphasised the necessity of players communicating with their coaches and avoid playing with pain. Balance between exercise and recovery, sufficient sleep and appropriate diet were acknowledged as important player wellness factors. Coaches described that players can put themselves at risk by continuing to play despite pain, being poorly trained, not performing sufficient rehabilitation or having a risk-taking playing style. Overload, playing surfaces and equipment, as well as maturation and player sex, were seen as potential risk factors for pain.

**Table 2:** Examples of quotes for the four subcategories in the main category: *Can pain be avoided?*

ID	Meaning unit	Subcategory
5	We do basic training to get the guys in shape, so that they can handle higher external loads. But we don’t have any specific exercises to prevent pain.	Coach strategies to reduce the risk of pain
12	We really stress this; no one shoots a single shot before training starts and before they have warmed up properly. It’s okay if you come out early and run and do easy ball exercises. You jog a bit, pass a bit, and start your Knee Control and then warm up your whole body. You do that before […] football. It’s not negotiable.	
17	As a player you can listen to your body. You can tell the coach ‘we’re going too hard this week’. It’s important that you’re open and honest as a player, so you don’t push yourself too hard. You don’t want to show yourself as being weak for the team, often you push yourself too hard […], it’s a game and then you play anyway.	Player actions to prevent pain
19	It is very much in your own interest how you recover afterwards. What do you do when you get home? Do you drink enough water? Do you eat enough? Do you sleep enough?	
3	I know there are those who have played when they have pain. They know that we coaches don’t let them play an important match if they have pain. So, they don’t say anything. Then, you notice it after they have played 20 minutes, and they come out and have a lot of pain instead.	Players ignoring risks
12	Some players are involved in more clashes depending on where they play on the field. Some expose themselves to more, they play more physically, sometimes maybe on the edge. Then both they and the opponent can get injured. So, it depends a bit on how you act.	
8	Everything becomes faster in football. The demands players place on themselves and on others get higher all the time.	Demands of the sport
5	We will increase to three football sessions per week soon. Then it becomes quite high load for them during pre-season. It’s hard training, it’s high tempo, because now we’re going to do strength and conditioning. Then you get sore muscles, and you get a little pain here and there.	

ID refers to the coach ID within the study. Some quotations were shortened, which is indicated by […].

### How to manage players with pain?

Coaches described pain management strategies in three subcategories*: coach actions to manage pain, positive communication climate* and *use of pain medication* (table 3). Coaches assessed and treated various complaints and pain, including acute injuries. Coaches emphasised the need for proper education and access to medical competence to manage players with pain adequately. They said they value collaboration with coach colleagues and medical personnel since various competencies are needed to work with players. Coaches demonstrated a genuine concern for pain management and encountered challenges in interpreting and effectively addressing diverse pain conditions. Coaches valued good coach–player relationships, having fun in the team and open discussions. They emphasised the importance of paying attention to all players and responding to any signs of pain. They stressed effective collaboration, shared responsibility between coaches and players, and the importance of players being comfortable reporting pain. Coaches were uncertain about players’ consumption of oral and topical drugs. Still, they believed pain medication was rarely used in amateur football, except for occasional non-sport-related or football-related pain or in important matches with a player shortage.

**Table 3:** Examples of quotes for the three subcategories in the main category: *How to manage players with pain?*

ID	Meaning unit	Subcategory
12	One […] sees the situation that happens and what can come of it. There is always a medical bag nearby. There is the possibility of applying pressure and cold and such things.	Coach actions to manage pain
10	If a player says, “my knees hurt when I do this exercise,” then I would say “you shouldn’t do anything that hurts, maybe you can still do the exercise but not as deep?”.	
20	When players experience pain, we always ask them to call the physiotherapist so he can make an assessment.	
8	Pain management should be emphasized more on training courses and such. You could have a physiotherapist who can educate on this topic.	
10	We have been open and talked a lot about menstruation, everything between heaven and earth, with the girls. We think it is so important. It has not only been football, but it has been about their whole life. We have talked about the body and about menstruation, that you can have a stomach-ache and sometimes it is more comfortable to train anyway. They have absolutely been able to come to us and tell us if they have pain.	Positive communication climate
16	We have developed guiding words; a culture code, I usually call it. Our guiding words are that we should have fun, dare to have courage, and dare to be honest. We should talk in a positive way. To be good people simply, both coaches and players.	
3	I know that many of these girls use it during their period, the last few years there have been a lot of menstrual pains. It is really difficult, because then maybe they take Paracetamol and so on during these days and it is not related to football at all, but they train with painkillers. […] But I don’t think anyone who has an injury takes painkillers to train.	Use of pain medication
5	I don’t know, really. It’s possible that someone sometimes takes painkillers before a match because they have pain. It’s nothing I know of. But I can imagine that some do it because they really want to play.	

ID refers to the coach ID within the study. Some quotations were shortened, which is indicated by […].

### What resources do we need?

Coaches described various prerequisites for pain management in four subcategories: *club resources and intentions, coaches need competence to manage pain, support from medical resources* and *parental support for youth players* (table 4). Coaches emphasised the importance of support from the association and club. They highlighted the need for specific competencies and resources for players, adequate coach education and access to medical personnel. Coaches stated that players who return to play after injury may have incomplete recovery, require coach supervision and training adjustment for pain-free rehabilitation, and return to team training without medical staff.

**Table 4:** Examples of quotes for the four subcategories in the main category: *What resources do we need?*

ID	Meaning unit	Subcategory
9	[Do you have any routines within the club for taking care of players who have pain?]No, I wouldn't say we have. It’s probably restricted to what we know ourselves.	Club resources and intentions
19	I think there are huge shortcomings [in routines to take care of a player who has pain and who is going to return to team training after rehabilitation]). […] If you look at these smaller clubs, they don't have that expertise or knowledge. You may not have the time, either.	
4	I feel like, personally, I would like to have more education about how to deal with injuries and deal with pain. I think players would also benefit from getting more info from people who are good at different types of injuries. As well as getting information on how to handle pain and how to think about pain.	Coaches need competence to manage pain
13	They usually ask for advice. Sometimes you feel like you're a kind of hobby physiotherapist, and you don't want to be that. You don't want to advise them to do anything wrong. […] It’s very hard to know what to do instead. Maybe these little rehab pieces that you would like to get in. Sometimes you can join in and train, but you would like them to get an extra rehab or rest an extra day. I find those parts most difficult.	
4	If there is something that he [a physical trainer and rehab manager in the team who is a chiropractor] thinks they should proceed with, he will refer to a clinic for further consultation and possible rehab. […] So that they get a 100% correct rehab program from a physiotherapist.	Support from medical resources
6	There was one who came to me, a goalkeeper who had dislocated his finger, it was going in the wrong direction. He wanted me to twitch a little and push it back. That time I said no, we'll take you to the emergency room instead. That kind of thing happens sometimes. They come and want me to do it. It’s incredible.	
2	Then [when a young player with a pain condition is going to return to sports after completing rehabilitation] it is important to have parental support. If you have interested parents, or parents who have played football, then it’s easier to be able to help a little.	Parental support for youth players
1	If it’s a youth team, you have parental contact. I would never just talk about what children should do without talking to the children’s parents or guardians. It’s very important. When they turn 18 and come of age, then you can address it right away. […] Maybe you should go to the health centre for a check.	

ID refers to the coach ID within the study. Some quotations were shortened, which is indicated by […].

## Discussion

The study showed that coaches viewed pain as a warning sign that should be adequately managed, and their attitude towards pain medication was restrictive. Coaches believed that certain pain can be prevented and that prioritising preventive measures is crucial. The absence of pain was intricately linked to sports performance and players’ long-term sustainability. Coaches’ keys to reducing the risk of pain included structured and customised training, a well-balanced training load and recovery, and a positive communication climate in the team. They emphasised the need for education and support for coaches and access to medical competence.

### Interpretation of pain

This study focuses on pain during sports participation, embracing various types of pain that may be present during football play, including pain related to acute and gradual-onset injuries, overload and other types of pain, such as menstrual pain and headaches.^8 9^ Coaches expressed different pain thresholds and various interpretations of pain in their players. They experienced that some players manifest pain signals readily, while others hide them and continue to play despite potential serious injury. Pain is a complex and subjective phenomenon that various factors, such as psychological traits, cultural learning and the meaning of the situation, can influence.^20^ While viewing pain as a potential sign of injury, coaches acknowledged the complexity of interpreting pain signals and assessing injury severity for decisions on the need for further management by health professionals. Coaches recognised that they cannot rely on the player’s pain perceptions alone due to individual variations in pain thresholds and expressions. However, by developing a deeper understanding of their players, coaches believed they could enhance communication and facilitate effective pain management. This aligns with current knowledge that psychological factors need to be considered to promote recovery from sports-related pain and optimise return to play.^21^

### Strategies to limit and manage pain

Appropriate training load and sufficient recovery were crucial factors to mitigate pain among players. Coaches recognised the significance of wellness factors, including adequate sleep and nutrition, while emphasising the players’ responsibility for self-care and maintaining their physical well-being.

A positive communication climate was key to efficient pain management within the team. This aligns with a recent systematic review that identified important variables for team function and performance applicable in sports: leadership styles, supportive team behaviour, communication and performance feedback.^22^ In professional football, efficient leadership behaviour impacts players and teams by establishing an interpersonal environment that supports, respects, trusts and appreciates staff and players.^23^ Likewise, in the present study, coaches perceived that a communication climate characterised by openness, trust, support and respect fostered team cohesion and performance. Coaches valued parental support for youth players with pain and highlighted the importance of a dialogue involving the athletes and their caregivers.

Coaches underscored the importance of medical expertise as a fundamental component of effective pain management strategies. This aligns with the literature showing that access to sports medicine clinicians is a prerequisite for adequately diagnosing and treating sports-related pain disorders.^13^

### Views on and use of pain medication

Coaches demonstrated a cautious view regarding pain medication. They perceived its use as relatively infrequent but acknowledged their limited awareness of players’ pain medication consumption. Pain medication, especially non-steroidal anti-inflammatory drugs (NSAIDs), is reported to be commonly used in elite^16^ and youth^24^ athletes. However, the prevalence of NSAID use is lower among non-elite youths.^24^ In young elite athletes, higher pain medication use is seen in athletes with greater willingness to compete while injured, which implies that pain medication may be an ingrained part of a sport-specific culture of risk acceptance.^25^ Coaches experienced that topical drugs were more commonly used than oral drugs. A recent systematic review recommended that athletes use topical medications for pain reduction since topical medications are more effective in reducing pain and are associated with fewer adverse effects compared with oral medications.^26^

## Clinical implications

Adequate pain management is important and should be based on the physiological, anatomical and psychosocial impacts of an individual’s pain.^27^ This study illustrates that amateur football coaches often assume great responsibility in guiding players through pain during sports participation, including assessing whether players experiencing pain can participate in team training and match play, individual adaptions of the team training and deciding when referrals to medical personnel are necessary. Amateur coaches work closely with their players and are crucial stakeholders and effective conduits for promoting health strategies and pain prevention within the sport. This study highlights a need for education and ongoing support for coaches to work strategically to reduce the occurrence of pain in their players and to be able to handle players with pain. Educational efforts and guidelines for pain management should be provided in all clubs. Training load and recovery, communication, injury prevention and pain management are important areas to address.

## Limitations

One limitation is that the interview consisted of two parts: implementation of injury prevention exercise programmes and pain in sports. Coaches sometimes associated pain with injuries and management of pain with injury preventive measures, and the double focus in the interview may have stimulated these lines of thought. When interpreting the study findings, it should be noted that all participating coaches in this study had prior experience with *Knee Control/Knee Control+* and willingly agreed to be interviewed regarding pain management and injury prevention. Therefore, these coaches may be particularly interested in health and pain management compared with coaches in general. To assess the trustworthiness of the study, we address the criteria of credibility, transferability, dependability and confirmability originally proposed in the framework authored by Guba and Lincoln^28^ by adopting strategies presented by Shenton.^29^ To enhance the credibility of our study, we conducted a negative case analysis. Specifically, we actively sought data that did not align with the existing categories. This collaborative process involved all three researchers, allowing for peer scrutiny and validation.^29^ To facilitate transferability, sufficient information regarding the study context is presented, enabling readers to assess the degree of similarity between the prevailing environment and other settings.^29^ We included coaches from both male and female teams, and from both small and large clubs. Only 2 of the 20 coaches were female, which reflects the sex distribution among football coaches at this level. The results are likely transferable to similar contexts, predominantly amateur football, with coaches working voluntarily, although they may not be universally applicable. Since qualitative content analysis was employed, data saturation is commonly not discussed. However, the 20 interviews with coaches representing different perspectives were perceived as sufficient to respond to the study’s aim. During the analysis process, we assessed that the actual sample held adequate information power to support the formation of new knowledge.^30^

We provided detailed methodological descriptions to enhance dependability and confirmability, including transparent and systematic procedures to minimise bias and subjectivity. Peer triangulation among researchers ensured neutrality in interpreting findings. After completing the analysis, we systematically reviewed the interview data to ensure that the overall findings accurately represent the content elicited during the interviews.^29^ In terms of reflexivity, self-reflection by the research team assumes significance.^31^ Our professional backgrounds as sports physiotherapists could influence participants’ responses to the interview questions. Therefore, the interviewer introduced herself as a researcher. A significant barrier that can keep participants from expressing their opinions could be a perception that the researcher favours particular views.^32^ To address this challenge, the interviewer strategically established a foundation of trust with the respondent. This involved emphasising the value of the participant’s experiences and thoughts throughout the interview process.

## Conclusion

Coaches’ perceptions of pain during sports participation and approach to pain management emerged as one theme: *To play or not to play—coaches navigating difficult terrain with limited resources*. The theme reflects that coaches must assume great responsibility despite limited medical competence. Coaches believed that adequate pain management is important, and their keys to reducing the risk of pain included structured and customised training, a well-balanced training load and recovery, and a positive communication climate in the team. Coaches often decide whether players with pain can participate in team training and match play and emphasise the need for education and support for coaches and access to medical competence.

## supplementary material

10.1136/bmjsem-2024-001941 online supplemental file 1

## Data Availability

All data relevant to the study are included in the article or uploaded as supplemental information.
