# Supplementary material for: To play or not to play, that is the question: an interview study with amateur football coaches on perceptions of pain during sports participation
Source: BMJ Open Sport Exerc Med. 2024 Jul 9;10(3):e001941. doi: 10.1136/bmjsem-2024-001941 (PMC11243123; doi:10.1136/bmjsem-2024-001941)
Supplement: online supplemental file 1 [file bmjsem-10-3-s001.pdf]

# Appendix
# Interview guide

1. Please describe your experiences of pain in relation to football.

2. How do you handle a player with pain in your team/club?
    a. Do you have any routines or support in your club or team on how to handle players who get injured (acute injury versus gradual onset injury)?

3. Do you think there is anything you can do to avoid pain when playing football?
    a. What can the player do?
    b. What can the coach do?

4. What are your thoughts on pain medication when playing football?
    a. In your experience, how common is it that football players use pain medication?
    b. If football players use pain medication, why do you think they do it?
    c. What are your thoughts on the coach's role?

Final question

5. Do you have any additional thoughts on pain and football that you want to tell us about?

Follow-up questions

• Can you explain more?

• Can you give an example?
